# The East and Southern Africa Ministerial Commitment: a review of progress toward fulfilling young people's sexual and reproductive health and rights (2013–2018)

**DOI:** 10.1080/26410397.2021.1982186

**Published:** 2021-11-02

**Authors:** Katherine Watson, Elsie Akwara, Patricia Machawira, Maria Bakaroudis, Renata Tallarico, Venkatraman Chandra-Mouli

**Affiliations:** a Research Consultant, World Health Organization/Human Reproduction Programme, Singapore.; b Research Consultant, World Health Organization/Human Reproduction Programme, Geneva, Switzerland; c Regional Health Education and HIV Advisor, UNESCO Regional Office for Southern Africa, Harare, Zimbabwe; d Comprehensive Sexuality Education Specialist & Disability Focal Point, UNFPA East and Southern Africa, Johannesburg, South Africa; e Youth Team Lead and SYP Regional Coordinator, UNFPA East and Southern Africa, Johannesburg, South Africa; f Scientist, World Health Organization/Human Reproduction Programme, Geneva, Switzerland

**Keywords:** adolescents, young people, sexual and reproductive health and rights, East Africa, Southern Africa, policy

## Abstract

Whilst significant progress has been made in recent years with respect to advancing health and education, the ESA region’s young people still experience challenges in relation to their SRHR. This study brings together learning from the available data and reports, as well as the experiences of those closely connected with the process of affirmation/endorsement and implementation of the ESA Commitment between 2013 and 2018. Whilst challenges remain – particularly in relation to monitoring and accountability – the ESA Commitment has instigated notable progress, made possible in part by the emphasis on multisectoral collaboration between health and education sectors nationally and regionally. Sustained political, technical, and financial investment in young people’s health and rights will ensure that the countries of the region are able to build upon these successes and deliver on their commitments to young people during this decade of action towards the realisation of the SDGs and harnessing the demographic dividend for Africa for years to come.

## Résumé

Si des progrès importants ont été accomplis ces dernières années en matière de santé et d’éducation, les jeunes de la région de l’Afrique orientale et australe rencontrent encore des obstacles en rapport avec leur santé et leurs droits sexuels et reproductifs. Cette étude rassemble des enseignements tirés des données et rapports disponibles, ainsi que des expériences des personnes étroitement liées au processus d’affirmation/approbation et mise en œuvre de l’engagement ministériel de l’Afrique orientale et australe entre 2013 et 2018. Si des écueils persistent, en particulier en relation avec le suivi et la redevabilité, l’engagement ministériel a stimulé des progrès importants, rendus possibles en partie par l’accent placé sur la collaboration multisectorielle entre la santé et l’éducation aux niveaux régional et national. Des investissements financiers, techniques et politiques suivis en faveur de la santé et des droits des jeunes permettront aux pays de la région de se fonder sur ces succès et d’honorer leurs engagements envers les jeunes pendant cette décennie d’action, dans la perspective de la réalisation des ODD et l’exploitation du dividende démographique pour l’Afrique pendant les années à venir.

## Resumen

Aunque en los últimos años se ha logrado progreso significativo con respecto a la promoción de la salud y la educación, la juventud en la región de África oriental y meridional aún enfrenta retos con relación a su SDSR. Este estudio reúne conocimientos adquiridos de los datos e informes disponibles, así como las experiencias de las personas conectadas estrechamente con el proceso de afirmación/aprobación y aplicación del Compromiso adquirido por la región en 2013 y 2018. Aunque aún hay retos, en particular con relación al monitoreo y la rendición de cuentas, el Compromiso ha instigado progreso notable, que en parte ha sido posible debido al énfasis en la colaboración multisectorial entre los sectores de salud y educación a nivel nacional y regional. La continua inversión política, técnica y financiera en la salud y los derechos de las personas jóvenes garantizará que los países de la región puedan basarse en estos logros y cumplir sus compromisos con la juventud durante esta década de acción para realizar los ODM y aprovechar el dividendo demográfico para África durante muchos años.

## Introduction

**Figure 1.**
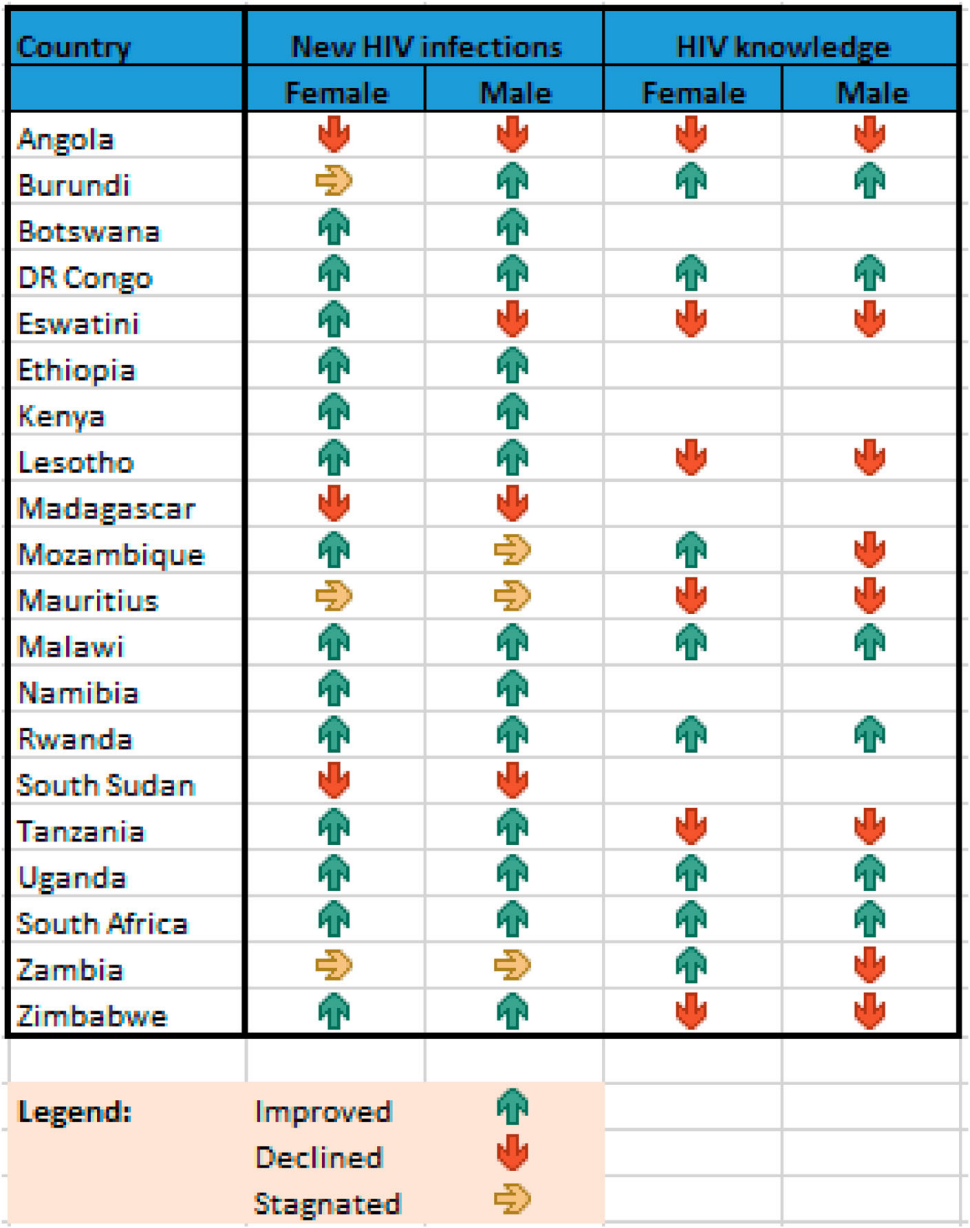
New HIV infections and comprehensive knowledge of HIV among young people 15 to 24 years old . Data source: AIDSInfo 2013–2018 data. http://aidsinfo.unaids.org/; 2010–2018 DHS, MICS, AIS reports

**Figure 2.**
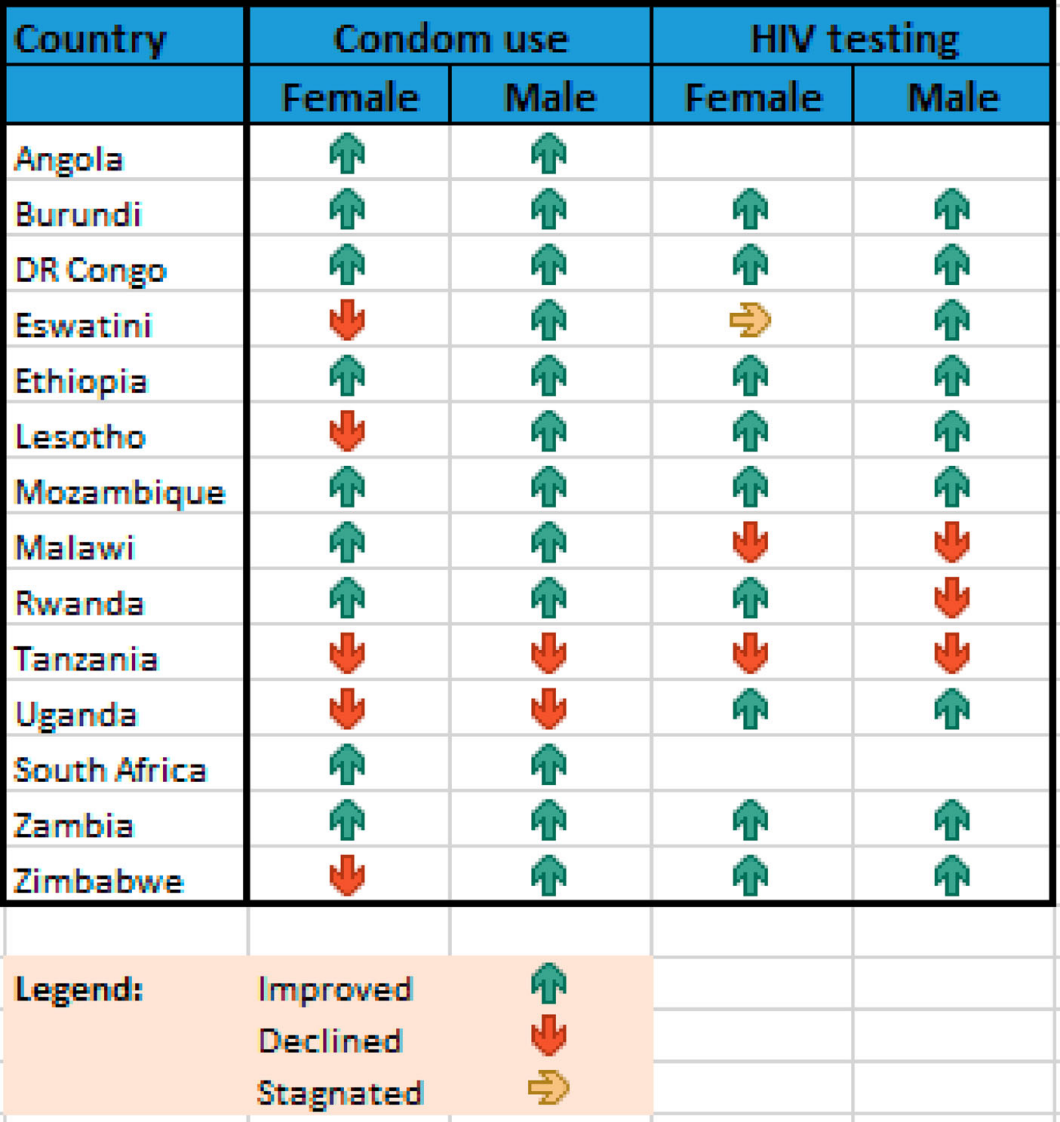
Condom use and HIV testing behaviour among young people aged

The Eastern and Southern Africa (ESA) region is home to more than 165 million young people,* defined as those aged 10–24; this figure is expected to climb to 263 million by 2050.^1^ Although significant progress has been made in recent years with respect to advancing their health and education, the region’s young people still experience challenges in relation to their sexual and reproductive health and rights (SRHR). Whilst there is a noticeable, positive upward trend in HIV knowledge levels, less than 40% of young people in the ESA region have sufficient knowledge about HIV prevention; at the same time, 2.2 million young people aged 15–24 are living with HIV.^2^ Adolescent† fertility rates remain persistently high at 85.9 and 66.9 live births per 1000 girls aged 15–19 in the region, in Eastern Africa and Southern Africa, respectively, a statistic higher than the world average of 39.9.^3^ The availability of these and other data documenting the impact of HIV, child marriage, early and unintended pregnancy and gender-based violence (GBV) helped to thrust young people’s SRHR to the top of governments’ political agendas across the ESA region during the first two decades of the twenty-first century. This collective regional momentum culminated in 2013 in the Ministerial Commitment on comprehensive sexuality education and sexual and reproductive health (SRH) services for adolescents and young people in Eastern and Southern African (ESA Commitment) (Figures 1 and 2).

This paper seeks to situate the ESA Commitment within a historical context, document the journey toward its affirmation/endorsement, and provide insights into progress and lessons learnt during the first five years of implementation. The affirmation/endorsement and implementation of the ESA Commitment were and continue to be complex processes involving actors at sub-national, national, regional and international levels. This study, therefore, is not exhaustive; rather, it provides “big picture” analysis from the perspectives of a handful of key organisations and individuals. The following questions were used to guide the research and analysis for this paper:

1. What was the context within which the ESA Commitment was developed?
2. What was the process for affirming/endorsing the ESA Commitment?
3. How was progress measured during the first five years of implementation?
4. What progress was made in the first five years of implementation?
5. What helped and hindered the ESA Commitment’s implementation during the first five years?

It should be noted that the first two questions focus on the context and political processes underlying the affirmation/endorsement and implementation of the ESA Commitment. Such process-oriented findings often go undocumented, and lessons learnt, that could be of use to others as they embark on similar advocacy and consensus-building efforts, remain stored away in the memories of the actors and institutions involved in these processes.

## Methods

Before the formal research began, the authors held conversations with several key actors in the ESA region to define the research questions. Thereafter, evidence for this study was collected in two phases, both of which included document review and key informant interviews (See Box 1). All the available evidence was reviewed in a systematic manner utilising the research questions as the organising schematic.

**Box 1:** Methods used to collect data for each research question

Research question	Data collection methods
*What was the context within which the ESA Commitment was developed?*	Document review Key informant interviews
*What was the process for affirming/endorsing the ESA Commitment?*	Document review
*How has progress been measured during the first five years of implementation?*	Document review
*What progress has been made in the first five years of implementation?*	Document review Key informant interviews
*What has helped and hindered the implementation?*	Document review Key informant interviews

### Literature review

The literature review was conducted using Google Scholar, PubMed, Cochrane Database and MEDLINE with the following key words and phrases: ESA Commitment; adolescent sexual and reproductive health (ASRH) in Eastern Africa; ASRH in Southern Africa; comprehensive sexuality education (CSE) in Sub-Saharan Africa; CSE in Eastern Africa; CSE in Southern Africa; youth-friendly services (YFS) in Eastern Africa, and YFS in Southern Africa. Thereafter, a “snowball approach” was utilised, whereby bibliographies included in the selected articles were also scanned for other relevant articles. Key experts were asked to submit relevant documentation as well. The inclusion criteria for the documents collected were:

- document directly addresses the research questions set for the study; and
- document covers the time period directly leading up to the affirmation/endorsement of the ESA Commitment (2008–2013) and/or the first five years of implementation (2013–2018).

A total of 17 documents were collected and all were reviewed; 14 of these were categorised as “grey literature”, whilst three were from peer-reviewed journals. The majority of the grey literature emanated from United Nations partners supporting the ESA Commitment. In order to systematically review the literature, a template organised around the five research questions was developed. Information relevant to the research questions was drawn out and, from that, themes were identified.

A further search for literature was carried out once the key informant interviews were finished in order to fill gaps in the available evidence and to fully answer the research questions. The same searches and inclusion criteria applied; given the time lapse, these were run to ensure that the most up-to-date publications were included. These later searches yielded an additional five peer-reviewed articles, as well as six documents considered to be “grey literature”. These documents were analysed in the same manner.

### Key informant interviews

Following the literature review, a standardised interview guide was developed and used with a total of 13 key informants who were directly involved in the development and/or implementation of the ESA Commitment and were purposefully selected by the authors. The first round of interviews included three government officials (Zambia, Eswatini, Malawi), two Southern Africa Development Community (SADC) officers, and four non-governmental organisation (NGO) representatives (AIDS Accountability International, Regional Psychosocial Support Initiative (REPSSI), AfriYAN, and Eastern Africa National Networks of AIDS and Health Service Organizations (EANNASO)). Four of these interviews were conducted on the phone and the other five interviewees responded in written format by email. Where needed for clarification purposes, the authors followed up with those who responded in writing. Following the analysis of information from the literature review and first round of interviews, the authors identified the need to conduct additional interviews. Four additional phone interviews were conducted with representatives of the High Level Group and UN agencies, using an adapted version of the interview guide that included questions that directly addressed the gaps in the research. All participants consented to take part in the study and agreed that the information provided could be cited in reporting, provided no personal, identifying information was shared. Similar to the literature review, a template was developed to analyse the comprehensive notes taken during all 13 interviews; thematic coding was utilised that allowed the authors to identify recurring themes for each research question.

Once the analysis of both the literature and the interviews were complete, the themes identified were reviewed, discussed and synthesised in an iterative manner by all authors. The “Results and discussion” section presents these themes and is organised by the original research questions.

## Results and discussion

### What was the context within which the ESA Commitment was developed?

During the first decade of the 2000s, a range of factors both inside and outside the region contributed to an emerging consensus around the importance of SRHR and CSE for young people. The evidence available for this study highlighted four contextual factors in particular that led to an ESA regional ministerial commitment. First, more and better data on the scope of the problem emerged, much of which highlighted the disproportionate impact of HIV, GBV and early and unintended pregnancy on young people. Second, in light of the HIV epidemic in the region, there was a burgeoning consensus on the need for multisectoral solutions, and the 2008 Mexico Declaration in the Latin American and Caribbean (LAC) region provided inspiration in this regard. Third, existing international, regional and national level commitments to CSE and SRHR paved the way for governmental support for the ESA Commitment which, in turn, provided civil society and youth movements with leverage for advocacy and accountability efforts – the fourth factor.

#### New evidence on the need for SRH services and CSE

Evidence published in the early part of the twenty-first century highlighted the adverse health and other outcomes experienced by young people in the ESA region. High rates of early and unintended pregnancy, GBV, child marriage and HIV infection rates alongside evidence of young people’s low levels of knowledge about HIV and modern contraception painted a concerning picture. In the year before the ESA Commitment’s affirmation/endorsement, it was reported that at least 20% of young women in six countries‡ in the region had started childbearing by the age of 17.^4^ In relation to GBV, between 15% and 35% of women reported having experienced sexual violence at some point in their lives in the nine countries for which data was available.^4^ The same year, UNFPA estimated that 34% of women aged 20–24 years living in the ESA region were married or in union by the age of 18.^5^ In 2012, an estimated 2.6 million young people aged 15–24 were living with HIV in the ESA region, with the overall prevalence rate amongst young women being over two times higher than among young men of the same age.^4^ The region was considered the “epicentre” of the HIV epidemic, and15 of UNAIDS’ priority countries were located within it.^6^

At the same time that the evidence base on young people’s SRHR expanded, two important bodies of evidence emerged on CSE. The first linked CSE with various SRH behavioural outcomes, including evidence to support that CSE was found to delay sexual activity, as well as to increase contraceptive usage amongst sexually active young people.^4^ The second body of literature spoke to the limitations of how CSE was delivered globally, regionally and in turn implemented at national level. In the years leading up to the ESA Commitment, the literature drew attention to the gaps between national sexuality education curricula (also known as “life skills” curricula and other names) and international standards, known as the UN International Technical Guidance on Sexuality Education (2008). Research published in 2010 found that, on average, 36% of Grade 6 pupils from the ESA region met the minimal knowledge standard on the HIV AIDS Knowledge Test, whilst just 7% achieved desirable knowledge levels.^7^ In 2012, a report commissioned by UNESCO and UNFPA on reviewing the curricula in 10 ESA countries found that, in general, curricula were lacking a range of topics related to SRHR and contained contradictory messages about gender and human rights. Whilst some curricula – such as the “Window of Hope” curriculum in Botswana and Namibia – included rights-based messaging, many others focused on abstinence-only approaches and employed fear-based messaging about sex, both of which are known to be ineffective.^8,9–10^

Almost unanimously, key informants identified the importance of data in mobilising and uniting actors at all levels, from civil society to UN agencies to high-level political actors. An NGO representative interviewed spoke of the importance of data on HIV and early and unintended pregnancy among adolescents, in particular, saying that these were “major drivers” for the ESA Commitment. A UN representative interviewed pointed to statistics on HIV prevalence amongst young women and girls as particularly impactful and stated that against the backdrop of the evidence available, “*it was clear for us as … UN agencies … that something extraordinary needed to be done”.* The former UNAIDS Regional Director and eventual leader of the ESA Commitment’s High Level Group, Dr Sheila Tlou, stressed during her interview just how important data were in getting ministers “on side”:

“*Before we even called the ministers, a whole survey in the region was done – what’s the situation? Of teenage pregnancy? HIV knowledge levels? That’s what we were able to show them. I’m seeing 30% knowledge, for example … That convinced the ministers who were not aware of the problem.*”

#### Consensus on the need for multisectoral collaboration

As understanding of the magnitude of the challenges facing the region grew, so did an understanding of the interconnectedness of the solutions needed. The diagnostic report commissioned to kick-start the ESA Commitment initiative opens by highlighting the impact of limited education on health outcomes: “*The impact of limited educational opportunities and poor quality education on health and well-being is wide-ranging*” (p.14).^4^ Further evidence of the importance of coordination between ministries was drawn from the progress made in the Latin America and the Caribbean (LAC) region following the affirmation of the Mexico City Ministerial Declaration “Educating to Prevent”.^6^ The Mexico Declaration, as it is commonly known, marked a partnership between ministries of health and education across LAC, committing them to joint planning, implementation, monitoring, evaluation and follow-up in recognition of the synergies needed to reverse the HIV epidemic and to deliver CSE and SRH services to young people.^11^ At the five-year mark after affirmation, countries in LAC reported 24 policy changes for SRHR and were halfway toward the Declaration’s goal of reducing by 75% the number of government schools that did not provide CSE.^12^ A UN representative interviewed expressed the opinion that the emerging evidence was accompanied by a recognition of the complexity of the issues at hand:

*“What the ESA Commitment does [is it] addresses and takes into account the multiple, difficult dimensions of the issues at hand. This is a complex set of issues, the sensitivity, the inter-sectorality (a main driver), the need for high level political leadership, and a multi-stakeholder approach.”*

#### Reminder to governments of their existing SRH commitments

For most governments in the region, addressing adolescent SRHR was not a new endeavour; all had made substantive commitments years – decades even – before the ESA Commitment. At the global level, the 1994 International Conference on Population and Development Programme of Action adopted by 179 Member States recognised the importance of adolescents’ reproductive health, whilst calling on governments to take action for the fulfilment of those rights.^13^ At the turn of the twenty-first century, all UN Member States committed to advancing health utilising the Millennium Development Goal (MDG) framework, which included goals relating to gender equality, maternal health and HIV; eventually, targets relating to (adolescent) reproductive health were also added. Regionally, and in part to facilitate the achievement of the MDG targets, African Union (AU) heads of state adopted the Abuja Declaration in 2001, pledging to commit 15% of their annual budgets to improving the health sector^14^; shortly thereafter, more SRHR-specific commitments were made with the AU Continental Policy Framework on SRHR in 2006 and the Maputo Plan of Action in 2007.

Several key informants pointed out that the ESA Commitment did not put new issues on the table, but rather packaged issues in an integrated way and reinforced existing agreements around young people’s SRHR with the aim of keeping governments accountable. A Southern African Development Community (SADC) representative, for example, shared that these precursory agreements were “*used as guiding momentum to ensure CSE political support for ESA”*, whilst an NGO representative saw the ESA Commitment as a mechanism for “* … remind[ing] [countries] of their commitments towards ICPD, HIV 2020 targets, FP2020 targets”*.

#### Mobilisation and momentum generated by civil society

Civil society organisations, including youth-led organisations and youth activists, played a crucial role in highlighting the limitations of CSE and SRH services for young people in the years leading up to 2013. In 2011, for example, during the Mali Youth Summit, young people highlighted the shortcomings of their governments’ HIV prevention efforts and called for more resources, better laws, and more services for young people.^15^ An NGO representative interviewed highlighted how civil society responded to the growing evidence base around SRH and CSE as well as social taboos relating to youth sexuality by mobilising political support for the ESA Commitment. Young people played a leading role in holding their governments to account: “*young people were saying it was time to act – they felt it is time to act, so we are committed – they actually put pressure to act on those issues … *” (Government representative).

### What was the process for affirming/endorsing the ESA Commitment?

The political processes leading up to the ESA Commitment were long and complex, involving multiple institutions and individuals from across the region. It is impossible to map the thousands of decisions made and steps taken, though it is possible to pinpoint the key actors and milestone moments in the years leading up to the affirmation/endorsement.

#### Actors involved in the affirmation/endorsement

In 2011, a range of stakeholders launched an inclusive process for defining the way forward for CSE and ASRH in the ESA region.^16^ UNESCO, UNFPA, UNICEF, UNAIDS and WHO (hereinafter referred to as the UN partners) worked closely with regional economic commissions (RECs), governments and civil society organisations. Additionally, a High Level Group (HLG) composed of regional leaders was drawn together to drive the process toward affirmation/endorsement, including drafting the text for the Commitment, and a Technical Coordinating Group (TCG) was established to provide technical support to the HLG and to mobilise resources and support at the regional level.^6^ Table 1 gives a more detailed overview of the key players that were involved in the affirmation/endorsement process.

**Table 1.** Who’s who

Joint United Nations Programme on HIV and AIDS (UNAIDS)	This was the UN agency tasked with coordinating the ESA Commitment Initiative.
United Nations Educational, Scientific and Cultural Organization (UNESCO), United Nations Population Fund (UNFPA), United Nations Children’s Fund (UNICEF), World Health Organization (WHO)	These were the other UN agencies involved in the affirmation/endorsement and implementation of the ESA Commitment and that provided technical and financial support.
Southern African Development Community (SADC), Eastern African Community (EAC), COMESA (Common Market for Eastern & Southern Africa)	Regional economic communities (RECs) supported and liaised with governments throughout the ESA Commitment affirmation/endorsement and implementation stages and have been important partners in the accountability processes.
Federal Ministry for Economic Cooperation and Development (BMZ), Ford Foundation, Swedish International Development Cooperation Agency (SIDA), Swiss Agency for Development and Cooperation (SDC)	These are the bi-lateral donor agencies that have provided technical and financial support for the ESA Commitment affirmation/endorsement and implementation.
High Level Group (HLG)	Created for the ESA Commitment Initiative, the HLG is comprised of key regional leaders in SRH and education, young people and civil society with support from the RECs and the UN; it was led by Dr Sheila Tlou of UNAIDS and convened by UNESCO (on behalf of partners). The HLG steered the ESA Commitment process and played a key role in advising on the formulation of the final written commitment.
Technical Coordinating Group (TCG)	The TCG is a group of UN partners (UNFPA, UNICEF, WHO, UNAIDS and UNESCO), development partners (BMZ, SIDA, SDC, and USAID), civil society organisations (INERELA+, IPPF, Rebranding HIV) and Regional Economic Communities (SADC and EAC), the function of which is to provide technical support to the HLG and to assist in mobilising support and resources at regional and country level. There is also an expanded TCG, which included country representatives.
Civil Society Platform	CSOs were engaged from the outset. Their involvement was strengthened by the development of a CSO engagement strategy, which was based on the recognition of the need to systematically engage them in the realisation of the ESA Commitment in the region, and ensuring accountability as well as space and voice of youth at all levels. A regional coordination task team was established consisting of the following organisations: AIDS Accountability International, EANNASO, AFRIYAN, DHAT, SAT and SAFAIDS.

#### Timeline of the affirmation/endorsement

Table 2 provides an overview of the timeline leading up to the Commitment’s affirmation/endorsement and the start of implementation, by quarter. Simultaneous processes ran at the national and regional levels, ensuring the participation of all relevant stakeholders. Apart from the formalisation of various consultative bodies, the production of a diagnostic report was a crucial step; it provided the justification for the ESA Commitment and a baseline for measuring progress going forward.

**Table 2.** Timeline

Date	Event
Oct–Dec 2011	UNESCO, UNAIDS and BMZ launched the ESA Commitment Initiative High Level Group (HLG) and Technical Coordinating Group (TCG) established
Jan–Dec 2012	Health and Development Africa developed a diagnostic report commissioned by UNESCO that included evidence on HIV and sexuality education; accessibility of SRH services; rates of HIV and other sexually transmitted infections (STIs) amongst young people; and rights-based concerns related to gender inequalities, sexual identities and stigma and discrimination.
Jan–Mar 2013	UNESCO and Ford Foundation hosted a Southern Africa civil society consultation to review the diagnostic report HLG and TCG met face-to-face for the first time to review and endorse the diagnostic report
Apr–Jun 2013	UNESCO and IPPF hosted an Eastern Africa civil society consultation to review the diagnostic report HLG drafted recommendations for the ministerial commitment UNESCO with UNFPA drafted the text that ultimately formed the basis of the ministerial negotiations Country consultations took place in all twenty-one countries to validate the diagnostic report findings
Jul–Sep 2013	Country consultations continued in all twenty-one countries to validate the diagnostic report findings HLG advocacy with key policy makers in the region to garner support for the Commitment Diagnostic report launched at regional and national levels in October
Oct–Dec 2013	Twenty countries met and affirmed/endorsed the Commitment, which went into force on 7 December 2013.
Jan–Mar 2014	UN partners met and developed indicators for the regional accountability framework (RAF) based on data already collected in the region TCG tasked with monitoring implementation and developing progress reports for SADC and EAC Civil society monitoring platform created

### How was progress measured during the first five years of implementation?

Several processes were put in place to track progress at the regional level in relation to the ESA Commitment’s targets. The regional accountability framework developed by the TCG was intended to facilitate reporting to SADC and EAC, whilst also providing the basis for the monitoring efforts undertaken by other stakeholders.

#### Regional accountability framework

In affirming/endorsing the ESA Commitment, countries agreed to a set of nine common targets (see Table 3). In order to measure progress toward the achievement of these targets, the TCG developed a regional accountability framework (RAF) that included a set of 21 process and impact indicators. Data sources for the indicators included population-level surveys, UNAIDS annual estimates for the region, Education Management Information Systems’ Annual School Census, annual State of the World’s Children reports and – importantly – self reporting by countries at regular intervals using standardised templates.

**Table 3.** ESA Commitment targets

2015 Targets	2020 Targets
4.1 A good quality CSE curriculum framework is in place and being implemented in each of the 20 countries; 4.2 Pre and in-service SRH and CSE training for teachers, health and social workers are in place and being implemented in all 20 countries; 4.3 By the end of 2015, decrease by 50% the number of adolescents and young people who do not have access to youth-friendly SRH services including HIV that are equitable, accessible, acceptable, appropriate and effective.	4.4 Consolidate recent and hard-won gains in the reduction of HIV prevalence in ESA, and push towards eliminating all new HIV infections amongst adolescents and young people aged 10–24; 4.5 Increase to 95% the number of adolescents and young people, aged 10–24, who demonstrate comprehensive HIV prevention knowledge levels; 4.6 Reduce early and unintended pregnancies among young people by 75%; 4.7 Eliminate gender-based violence; 4.8 Eliminate child marriage; 4.9 Increase the number of all schools and teacher training institutions that provide CSE to 75%

The data collected for the RAF indicators were intended to be utilised in reporting to SADC and EAC – the regional accountability mechanisms for the ESA Commitment. According to key informants, the ESA Commitment became a standing item on the SADC ministers of education meetings every year after its affirmation/endorsement; however, there was no clarity amongst key informants about whether this was also the case for the SADC health ministers, who meet separately. There was no data available on how countries reported to the EAC against the ESA Commitment targets and indicators.

#### Reporting systems

The RAF provided a framework for the development of ESA Commitment progress reports published at regular intervals during the first five years of implementation. Four publicly available reports (see below) covering the first five years of implementation were published. Each report used its own methodology to collect and analyse data. None of the four reports presented data for all 21 indicators in the RAF.

- In 2015, the TCG, with support from the UN, published a brief report that presented, broadly, the progress that had been made since the affirmation/endorsement. Data presented related primarily to policy and budgetary RAF indicators.^17^
- In 2016, TCG partners published a progress report on implementation up to the end of 2015. It presented progress against the three ESA Commitment targets and reported that all had been met and exceeded. The report was based on country level reports which, in turn, were generated through national level consultations. Results were validated by national technical working groups, though not all members were present during the process.^18^
- In 2018, the Civil Society Platform, with support from the TCG, published a progress report developed through qualitative consultation with civil society organisations in Kenya, Lesotho, Malawi, Mozambique, Namibia, South Sudan, South Africa, Swaziland, Tanzania, Uganda, Zambia, and Zimbabwe.§ Data for the report were also collected through a questionnaire structured around nine RAF indicators and administered to government officials and other national level stakeholders.^19^
- In 2019, the TCG published a report covering progress up to the end of 2018 and presenting evidence of progress against each of the targets in the ESA Commitment using some of the RAF indicators. Data for the report was derived from population-based surveys; data collected by UNESCO in 2018 for its Our Rights, Our Lives, Our Future (O3) Programme; and responses to a questionnaire from UNESCO country offices in consultation with government officials and other national stakeholders. In addition, a draft report was validated through multi-stakeholder meetings in 12 countries.^17^**

### What progress was made in the first five years of implementation?

Tangible progress occurred in policy and programming for young people’s SRHR; however, these changes are not yet reflected in population-level survey data on adolescents’ lives.

#### Notable progress in policy and programme decisions, but unmatched with progress in population-level survey data

The data available for the RAF indicators point to progress against several ESA Commitment targets (Table 4),†† including policy-level, structural commitments, such as developing costed CSE strategies for out-of-school youth, setting up/revitalising multisectoral task teams and developing programmes to prevent child marriage. The unavailability of data for many indicators and a lack of comparability between countries are amongst the complexities that make it difficult to aggregate data and identify trends at the regional level. Further, for indicators that relied on population-level survey data – e.g. percentage of young women married before 18 and percentage of women who believe wife-beating is justified – the trends were not always in the desired direction.

**Table 4.** ESA Commitment progress

Indicators	Baseline	2015 Progress	2018 Progress	2020 Target	Explanatory comments
**Target : A good quality CSE curriculum framework is in place and being implemented in each of the 21 countries**^a^					
% of schools that provided life skills-based HIV and sexuality education in the previous academic year^18^^,^^19^^,^^20^	Not available	88% (Primary) 71% (Secondary)	14 out of 15 countries that reported on this indicator showed varying levels of coverage from 5% to 100%	95%	The 2015 and 2020 figures were extracted from the RAF, which states that the 2015 figure is based upon 2015 ESA Commitment country reports. The 2018 figure was taken from the 2018 report and was also based on country reports.
Number of countries that have a costed national CSE strategy or framework for out-of-school youth^18^^,^^19^^,^^20^	2 countries	Not available	17 countries	20 countries	The baseline, 2018 and 2020 figures were extracted from the 2018 progress report. The 2015 progress report presents three different figures for 2015. On page 33, it states that 16 countries have a costed strategy, whilst page 35 states that it is 15 in Figure 3.2. In the country annex, only 6 countries report having such a strategy. In addition, the RAF shows that 14 countries had a national CSE strategy or framework for out-of-school youth by the end of 2014. No data is presented in the table given these discrepancies. In the RAF, the indicator wording omits “costed“ and appears as “Number of countries with a national CSE strategy or framework for out of school youth.” However, the word “costed” appears in the 2015 and 2018 reports.
% of schools with teachers who received training, and taught lessons, in life skills based HIV and sexuality education in the previous academic year^18^^,^^20^	Not available	76% (primary) 56% (secondary)	Not available	90%	This indicator appears in the RAF and the 2015 report but not in the 2018 progress report. The data presented for 2015 is found in the RAF and is based upon the 2015 ESA country reports.
**Target: Pre- and in-service CSE and SRH training for teachers and health and social workers is established and being implemented in all 20 countries**					
Number of countries that provide pre-service and/or in-service training programmes on the delivery of CSE^18^^,^^19^	2 countries	Pre-service: 10 countries In-service: 20 countries	20 countries	20 countries	The baseline and 2018 figures were extracted from the 2018 report. The 2015 figures are from the 2015 report. The 2020 target is from the wording of the ESA Commitment target.
Number of countries that provided pre- and in-service training programmes on the delivery of AYFHS^18^^,^^19^^,^^20^	No baseline available	Pre-service: 10 countries In-service: 17 countries	21 countries	20 countries	The 2018 figure was in the 2018 report; the 2015 figures were in the 2015 report; and the 2020 target was in the RAF.
**Target: Decrease by 50% the number of adolescents and young people who do not have access to youth friendly SRH services, including HIV-related services that are equitable, accessible, acceptable, appropriate and effective**					
Number of countries with a costed national strategy/plan to improve young people’s access to youth-friendly health services aligned to regional/ international standards^18^^,^^20^	Not available	20 countries	Not available	20 countries	The 2015 figure was taken from the 2015 report, and the 2020 target was found in the RAF.
Percentage of health service delivery points that offer a standard / minimum package of adolescent/ youth friendly/sensitive health services^18^^,^^19^^,^^20^	Not available	65% of countries offer a standard minimum package of YFSRH services	Eswatini (74%) Kenya (23%) South Africa (100%) Tanzania (30%) Uganda (17%) Zimbabwe (20%)	Not available	The 2015 figure comes from the 2015 report, which includes a different wording of the indicator: “% of countries that offer a standard minimum package of youth friendly SRH services.” In the 2018 report, the wording switched back to that which appears in the RAF. In the 2018 report, figures were presented only for the few countries that were able to report on the indicator that year.
Percentage of adolescent girls and boys and young women and men aged 10–24 and living with HIV currently receiving antiretroviral therapy^20^	Not available	Not available	Not available	Increase by 75% of baseline	This indicator appears only in the RAF. The RAF erroneously refers to a “reduction by 75%” for the 2020 figure, rather than an “increase.” The wording has been changed in this table.
**Target: Eliminate all new HIV infections among adolescents and young people aged 10-24**					
Number of new HIV infections among adolescents and young people (15–24 years)^18^^,^^19^^,^^20^	430,000 (2013)	236,196 F 115,216 M (excludes Burundi, DRC, Ethiopia and Seychelles and includes Eretria)	293,570 (2017)	Reduction by 75% of baseline (*n* = 107,500)	The baseline, 2015 and 2020 figures were found in the RAF. The 2018 figure was found in the 2018 report. The RAF and 2015 report indicate that the source for the baseline figure was UNAIDS’ estimates from 2013. The RAF indicates that the 2015 figure is based on 2016 UNAIDS estimates. The 2018 figure is presented in the 2018 report as being derived from the 2017 UNAIDS estimate.
Percentage of never married women and men aged 15–24 years who had sexual intercourse in the past 12 months and used a condom at the last sexual encounter^19^^,^^20^	Not available	Not available	Amongst reporting countries, most reported an increase in condom use at last sex, except for Ethiopia, which reported a decrease; percentage increases varied from 39.2% to 72.5%	75% decrease from baseline	The 2020 figure was presented in the RAF. The 2018 data were found in the 2018 report and are presented as they appear therein.
Percentage of sexually active women and men aged 15–24 who have been tested for HIV and received results in last 12 months^18^^,^^20^	Not available	23% (M) 30% (F)	Not available	Increase by 75% of baseline	The 2015 and 2020 figures were found in the RAF. The source cited in the RAF for the 2015 figure is the State of the World’s Children Report( 2016). The 2015 progress report presents different data for 2015 – 36.4% for men and 58% for women (aged 15–24); the citation for this is the Global AIDS Response Progress Reporting 2015. The wording in the RAF for the 2020 target read “reduction by 75% of baseline.” It is assumed that this was a mistake, thus it has been changed in this table to read “increase.”
**Target: Increase to 95% the number of adolescents and young people aged 10-24 who demonstrate comprehensive HIV prevention knowledge levels**					
Percentage of AYP aged 15–24 years who have comprehensive HIV prevention knowledge^18^^,^^19^^,^^20^	41% M 35% F	45% M 42% F	40% (composite)	95% (composite)	The baseline figure is extracted from the RAF, which cites the 2014 State of the World’s Children report as the source. The 2015 figure is found in the 2015 report, with a citation explaining that it is based on DHS data for just 12 countries. The 2018 report mentions a composite figure of 36% for 2015. The 2018 value comes from the 2018 report; on page 17, the report states that 40% is a 2017 figure from UNAIDS that represents an increase from the 2015 UNAIDS figure of 36%. There is a discrepancy between the figures used in the 2015 and 2018 reports for this indicator. The 2020 target is taken from the ESA Commitment target wording. Although the ESA Commitment target mentions the 10 to 24 age bracket, all of the data used to report on this indicator are for the 15 to 24 age bracket. Different wording is used for this indicator in the RAF, which reads: ‘Percentage of young people aged 15–24 who both correctly identify ways of preventing the sexual transmission of HIV and who reject major misconceptions about HIV transmission.’
**Target: Reduce early and unintended pregnancies among young people by 75%**					
Number of countries implementing a national policy/ strategy on pregnant learners^19^^,^^20^	5 countries	10 countries	16 countries	20 countries	The baseline and 2018 figures are taken from the 2018 report. The 2015 and 2020 figures are taken from the RAF. There is a discrepancy in the reporting of the 2015 figure, which appears as ‘9’ in the 2018 report. The 2018 report uses a different wording of the indicator, adding the words “and readmission” to the end.
Percentage of women age 15–19 who have begun childbearing^18^^,^^20^	Births by age 18: 27%	Not available	Not available	Reduction by 75%	The baseline and 2020 figures were extracted from the RAF. The State of the World’s Children report (2014) is stated as the source for the baseline figure. A proxy indicator – births by age 18 – is used for the baseline. Data on this indicator in the 2015 report is presented separately for each of the 12 countries for which data are available; no aggregated figure is given.
**Target: Eliminate gender based violence**					
Number of countries whose education sector policies address school-related GBV^19^^,^^20^	7 countries	12 countries	18 countries	20 countries	All figures come from the 2018 report.
Percentage of women aged 15–24 who believe that wife-beating is justified for at least one of five specified reasons^19^^,^^20^	54%	52%	6.4% South Africa 13.3% Mozambique 18.6% Malawi 24.9% Angola 48.6% Zimbabwe 53.4% Uganda 59.4% Tanzania 60.3% Ethiopia	Reduction by 75% to 13.5%	The baseline and 2015 figures come from the RAF, which uses the following proxy indicator from the State of the World’s Children Report (2014 and 2016, respectively): ‘percentage of girls and boys 15–19 years old who consider a husband to be justified in hitting or beating his wife for at least one of the specified reasons, i.e., if his wife burns the food, argues with him, goes out without telling him, neglects the children or refuses sexual relations.’ The 2018 data is from the 2018 report, which presents data from the 8 countries in the region with population-level surveys administered after 2015. These data are for young women aged 15–24.
Percentage of education institutions that have rules and guideline for staff and students relating to physical safety, stigma and discrimination and sexual harassment and abuse that have been communicated to relevant stakeholders^20^	Not available	Not available	Not available	50%	This indicator is only included in the RAF and not in any progress reports.
**Target: Eliminate child marriage**					
Number of countries with programmes to prevent and mitigate against child marriage where prevalent^18^^,^^19^^–^^20^	Not available	12 countries	18 countries	20 countries	There are no baseline data available for this indicator. The figure in the 2015 column is from the RAF and the source is cited as the 2014 ESA Commitment country progress reports. The 2020 figure is also from the RAF. The 2018 figure is from the 2018 progress report.
Percentage of women aged 20–24 years who were first married or in union before age 15 and age 18^19^^,^^20^	10% by 15 years 38% by 18 years	10% by 15 years 36% by 18 years	Varies by country; significant progress in ESA but large disparities in child marriages between income quintiles, rural-urban divides, or by education levels	Reduction of 75% from baseline to .9% married by 15 and 3.6% married by 18	The baseline and 2015 figures are found in the RAF; the sources cited are the State of the World’s Children Report 2014 and 2016, respectively. The 2020 figures are also taken from the RAF. The 2018 report presents findings by country for 18 countries utilizing the latest DHS (or similar) survey data from each. Some of these surveys pre-date the ESA Commitment.
**Target: Increase the number of all schools and teacher training institutions that provide CSE to 75%**					
Not available	Not available	Not available	Not available	Not available	This target is found in the original, affirmed ESA Commitment but not in any other reports or documents. There were no indicators set for this target in the RAF.
**Target: Creating an enabling environment (not an original target within the ESA Commitment)**					
Number of countries implementing a multisectoral strategy or framework for operationalization of the ESA Commitment^19^^,^^20^	0 countries	18 countries	19 countries	20 countries	The baseline and 2020 figures are found in the RAF. The 2015 and 2018 figures are taken from the 2018 report.
Number of countries with a multisectoral task team established and functional to provide policy and technical guidance^19^^,^^20^	0 countries	14 countries	12 countries	20 countries	The baseline and 2020 figures are found in the RAF. The 2015 figure is also from the RAF but is from 2014. The 2018 figure is found in the 2018 progress report.
Number of countries having earmarked /mobilised financial resources for the implementation of the ESA Commitment^19^^,^^20^	0 countries	12 countries	12 countries	20 countries	The baseline, 2015 and 2020 figures are taken from the RAF. The 2015 figure, however, is from 2014. The 2018 figure is taken from the 2018 report.

^a^ Rwanda was the twenty-first country to affirm the ESA Commitment, although representatives from the country were not in attendance at the gathering during which it was affirmed.

#### Increased regional momentum and collaboration

Beyond the numbers, there was resounding consensus amongst key informants that the ESA Commitment has injected new energy into efforts to advance young people’s health, rights, and well-being at regional and national levels. Many commented on how, at the regional level, the ESA Commitment has created a united front for the advancement of CSE – still a difficult topic in many ESA countries. One NGO representative illustrated the importance of this, saying:

*“The ESA Commitment brought impetus to even us as civil society to push governments to do more around CSE in particular, but also access to services. For access to services, we had already made a lot of progress. Those adolescent strategies from UNFPA and from WHO – there was a lot of progress made and leverage there for accountability. When it came to CSE, all we had was the ITGSE [International Technical Guidance on Education] 2009. This gave us something at the regional level that was homegrown; our governments had committed, so was easier to push. I think to a great extent, it gave us enough power as civil society to push for CSE, whether it is us going into school directly or the government itself institutionalizing it.”*

Multisectoral collaboration is another area that the ESA Commitment is credited with revitalising. The 2018 progress report notes that “there has been a remarkable increase in the number of countries with a multisectoral strategy in place as the first step towards operationalizing the ESA Commitment”; indeed, by the end of 2018, 20 countries had such a strategy in place.^17^ Key informants recognised existing collaboration between sectors on the topic of SRHR; however, as one NGO representative put it:

*“The ESA Commitment didn’t create a separate group but, rather, work with existing structures and then ‘brand’ them as one of the TWGs [Technical Working Groups] for the ESA Commitment. That contributed to sustainability. In cases where this work was about to die, the ESA Commitment revamped it.”*

A government representative gave an example of how this multisectoral collaboration between health and education works, practically:

*“Since the Ministry of Education’s mandate is to impart knowledge and skills, we opted to share information and partnered with the Ministry of Health to offer services a hundred meters away from the school. The Ministry also opted to have a clear referral system wherein school youth are referred to a youth friendly health service center.”*

**Box 2: Multisectoral integration through the “Safeguarding Young People Programme”**

The Safeguard Young People programme has been implemented by UNFPA and its regional and national partners in eight Southern African countries‡‡ since November 2013. SYP is the only programme in the region that fully operationalises the ESA Commitment on CSE and SRH services for young people. The programme worked with the education sector in training educators on CSE; with the health sector in training health workers on youth-friendly services; with policy-makers in ensuring enabling legal environments; and with young people through networks at national and district levels, in finding ways to reach them more effectively. Notably, fifty-seven focus districts had functional referral systems between education, health and social service providers by 2020, and all eight countries had national CSE strategies in place for out-of-school youth.^18^

#### Anecdotal reports of progress in areas that were not measured

Beyond creating a united front and fostering multisectoral collaboration, individual key informants pointed to a host of other, unmeasured indicators of success. Some pointed to young people being more aware of their rights to CSE and SRH services, whilst others emphasised how the ESA Commitment has led to further investment in teacher and health service provider training schemes. There is also anecdotal evidence from key informants about the way that the ESA Commitment has inspired national CSE curricula reviews, integration of life skills into formal school curricula in primary and secondary schools, enhanced parental involvement and support for CSE, and heightened public awareness of young people’s rights across the region.

### What helped and hindered the ESA Commitment’s implementation during the first five years?

A number of lessons can be learnt from the first five years of the ESA Commitment’s implementation in relation to what has helped and hindered progress. High-level political commitment, regional ownership, youth leadership, and healthy competition amongst countries are all seen as factors that have advanced the ESA Commitment, whilst challenges relating to multisectoral collaboration, accountability structures, political turnover, sensitivities to CSE and financial resources were all identified as needing further attention.

#### Facilitating factors

The commitment of high-level political leaders has been a key enabling factor for the ESA Commitment’s implementation from day one. National champions such as the First Ladies of Malawi and Tanzania ensured political and governmental support, whilst also encouraging counterparts across the region to do the same. Likewise, champions within government ministries of health and education have grounded the ESA Commitment in the local realities. The 2018 progress report found that “leadership and ownership of the ESA Commitment by RECs and governments, including the establishment of national working groups, has been critical to its success”. (UNESCO 2019) One government representative echoed this sentiment, sharing that “*the political will amongst leaders and [their] support paved the way for successful implementation*”. One UN representative shared the importance of this high-level support in countering resistance to SRHR in the region:

“*Anything related to sexual and reproductive health rights – especially of young people – has been subjected in the past and unfortunately is still subjected to a strong opposition. UNFPA’s core mandate aims at ensuring universal access to SRHR for women and young people – therefore, we armed ourselves with all the necessary strategies to counteract any opposition. One of the strategies applied was the identification of high-level decision makers as champions of this initiative to work with us in ensuring than others* – *the ones to be converted – would agree to sign the commitment. This is the reason why UN Regional Directors and Regional Economic Communities became the backbone of the initiative. Their buy-in and advocacy efforts with Ministers in countries enabled action and gathered consensus.”*

This quote illustrates the importance of “buy-in” amongst a range of stakeholders, all of whom have influence over the realisation of young people’s rights. Involving the RECs and ensuring that they took leadership roles – e.g. in relation to accountability – was a strategy to ensure “*full ownership of the initiative and full alignment to regional priorities”* (UN representative). Ensuring that young people themselves were included as partners in all the processes was another such strategy. At the country level, for example, “* … the youth orgs that were part of the TCG would provide support to youth orgs who were part of the TWGs in the countries. Just to make sure that we’re speaking the same language”* (NGO representative).

A final facilitating factor identified by several key informants was the way in which the ESA Commitment created collegiality and collaboration between countries in the ESA region. One UN representative stated that the ESA Commitment “* … fostered a ‘healthy competition’ amongst member states to demonstrate achievements and innovation, also propelled by south-south cooperation and cross-country learning opportunities”.* Another key informant from an NGO explained the benefit of this positive peer pressure for advancing CSE:

*“The major benefit is peer pressure – seeing countries. I saw that a lot in ESA Commitment platforms; there was peer pressure from different countries. When you see what Zambia and South Africa are doing, it makes you want to reflect. We saw positive competition for success. A number of countries were working on CSE frameworks based on seeing other countries do it.”*

#### Hindering factors

Despite the increases in multisectoral collaboration across the region as noted in the previous section, there is evidence to suggest that sectoral divides persist. One report noted the “wasteful overlaps and duplication of activities” resulting from fragmented implementation between various government agencies.^17^^, p.57^ An NGO representative echoed this, stating that there is “weak collaboration” between health and education sectors, which are working in silos. The divides between education and health persist even at the level of SADC and EAC, as pointed out by one UN representative:

*“In the structure of SADC, as any organisation, we are structured … you are in departments. We seem to struggle with that. You get sectoral divides … that was the main reason to establish the ESA Commitment! You find the same challenges in SADC … they have ministerial meetings, the meeting of ministers of education/science/tech. We report back on ESA Commitment, but there is no joint annual meeting with health and education … they meet separately. I’m not sure whether health ministers discuss ESA Commitment.”*

Beyond these divides between health and education, other key informants lamented the lack of involvement of other ministries that work with and for young people. One NGO representative explained: *“Yes, it was important that health and education were in the same room, but the challenge was that youth at that time and even now are served by so many departments within government structure.”*

These divisions between sectors were also found in the monitoring and evaluation systems; given the lack of integration between government agencies, data was often hard to compile even within countries. This was documented in the 2018 progress report as a challenge in keeping governments to account for the achievement of ESA Commitment targets.^17^ There were further challenges with the accountability framework itself, with one NGO representative referring to it as “*an afterthought”*, explaining that the processes lacked clarity:

*“What I understood – ok, you have this commitment, and governments are accountable to SADC and EAC through those frameworks. They had to report annually. That wasn’t clear enough. First of all, when would they be reporting? What forum? Ministers of education? Or health? In which forum would it be discussed? When reports were being done, you could see that UN agencies were leading the processes, supporting countries to report. We weren’t then seeing SADC/EAC involved. Then, even their involvement, it was about individuals rather than institutional. The moment that the SADC person was un-funded, we no longer saw the person.”*

The lack of clear accountability processes also speaks to the need to institutionalise the ESA Commitment, rather than having it driven by individual champions, given the challenges presented by political turnover. A key informant from the UN who was emphatic about the importance of high-level political commitment, also pointed out the dangers of relying on this too much as a strategy for ensuring sustainable support:

*“I’m pointing at the strengths and weaknesses of the high level commitment. The strength is that it comes from the top. The weakness is that it has a shelf life – you need to nurture all over again. The case of Tanzania is telling – it went from strong support to now a situation where there is resistance.”*

A hindering factor noted by almost all key informants, as well as several reports, is the ideological resistance – particularly to CSE – emanating from both civil society and government. The 2015–2017 civil society perspectives progress report drew attention to pockets of resistance within communities, linking these with the slow progress on CSE.^17^ In Lesotho, for example, integration was met with opposition from religious leaders, parents, teachers, and community leaders who believed it would lead to early sexual debut and ran counter to traditional values.^17^ Another example of backlash is in Uganda, where the delivery of in-school CSE came to a halt whilst the government adapted the curricula, which no longer aligns with international standards. (UN representative) A government representative admitted that some countries’ ministers were not ready to accept “*all the issues, e.g. LGBTQ [lesbian, gay, bisexual, transgender and queer]”*. Similarly, two key informants from NGOs highlighted that it takes time to educate the general population on the value of CSE and to dispel popular myths, such as that CSE encourages young people to engage in sex.

Finally, there is much evidence to indicate that financial allocation during the first five years has been inadequate for implementation. Indeed, by the end of 2018, just 12 out of 21 countries had mobilised or identified financial resources for implementation.^17^ The 2015–2017 civil society perspectives report also highlighted the funding deficit, which is attributed to:

*“inadequate commitment (political or financial) from government; continually shifting and competing government priorities; heavy reliance on dwindling international aid; donor conditions; donor fatigue due to inefficiency, corruption, and mismanagement of funds; and lack of commitment for long-term funding for social change programmes.”*^17^

This same report called for increased domestic funding for ASRH to strengthen ownership and accountability.^17^ Key informants, too, noted the challenges posed by insufficient funds, chief amongst them the inadequate support of civil society and the engagement of organisations led by young people in the ESA Commitment’s implementation, monitoring and accountability processes.

## Lessons learned and ways forward

Through an examination of both processes and outcomes, this study draws out important lessons learnt for the advancement of the SRHR of young people in the ESA region, many of which may be applicable in other regions and for other causes. The findings indicate that the following three factors were crucial in bringing the ESA Commitment into being:

1. recent, reliable data highlighting the key challenges facing young people in relation to their SRHR to support evidence-based advocacy;
2. acknowledgement of the distinct contributions that a range of actors and sectors, including civil society, can make toward the advancement of common goals; and
3. building upon existing commitments and institutions (e.g. SADC and EAC) to encourage greater accountability and to foster local leadership.

Beyond affirmation/endorsement, the findings highlight the importance of the sustained engagement of all actors during implementation, particularly at a high political level. Given turnover within political systems, civil society – including youth-led organisations – plays a crucial advocacy role in ensuring that newly appointed representatives engage with existing processes and commitments. Situating accountability mechanisms locally, such as the ESA Commitment’s national working groups, brings accountability closer to home, promoting ownership and political will. At the same time, fostering an environment of healthy competition, South-to-South cooperation and learning at the regional level provides inspiration and motivation that can be harnessed by actors inside each country.

### Areas where stepped-up action is needed

Whilst the ESA Commitment contributed to notable progress and generated momentum, there is still a significant need for continued work to address remaining challenges such as those related to multisectoral collaboration, financing, opposition to CSE, measurement and accountability.

There is a need to continue to create spaces for collaboration between health and education that promote the well-being of young people. Despite the concerted efforts and progress made under the ESA Commitments, the long-standing divide between health and education persists in many countries, making practical collaboration challenging. This divide exists not only at a national level, but also at a regional level in the mechanisms and processes of the SADC and EAC as well as in the programming of civil society and UN partners. Alongside the need for collaboration between the health and education sectors, bringing on board other ministries, such as those concerned with youth development and gender, will help secure wider safety nets and harmonise efforts across all sectors that engage with young people.

Sustainable financing for ASRHR and CSE continues to be a challenge for most countries in the region. At a governmental level, additional funding – including blended financing – and institutionalisation are needed to ensure that commitments made in regional and international fora can take root. Further, the crucial roles that civil society – including organisations and networks led by young people – play in accountability efforts should be recognised, encouraged and funded.

Given the sensitivities related to CSE, continued advocacy and awareness raising on the benefits of CSE and SRH services for young people’s health and well-being and countering common myths with evidence are critical. This work must be sustained to garner, maintain and, ideally, increase political and public support for CSE.

Finally, creating a more effective monitoring and evaluation system based on the targets set by the ESA Commitment is necessary. The reliance on Demographic Health Survey (DHS) data for a number of indicators in the RAF meant that data from varying years were being compared. For other RAF indicators that relied on self-reporting by countries – e.g. percentage of schools that provided life skills-based HIV and sexuality education in the previous academic year – it is clear that not all countries were able to collect these data on a regular basis. Generally, there is a difficult balance to be struck between developing indicators for monitoring what needs to be monitored in order to demonstrate progress and ensuring that countries are not overburdened with data collection above and beyond what is absolutely necessary. At the same time, limitations on the type of progress that can be tracked and observed in short periods of time should be considered.

### Limitations

Though a careful review of the literature was carried out and key informants from across the ESA region were consulted, there were limitations to the study. Firstly, there were a very limited number of peer-reviewed publications available related to the research questions. As a result, the findings relied primarily on the grey literature and, in particular, ESA Commitment progress reports which, in turn, relied on self-reporting by governments and civil society organisations and were funded by UN partners involved in implementation. Second, the key informants disproportionately represented Southern African countries (i.e. there were fewer representatives from East African countries), though the literature filled in some of these gaps. The process of affirming/endorsing and implementing the Commitment has been and continues to be a long, complicated one with many actors involved across the 21 countries. The perspectives herein represent a small fraction of those who have been involved in and leading the efforts described. Finally, there were limitations on the indicators available to measure progress at the regional level.

## Conclusion

Whilst significant progress has been made in recent years with respect to advancing health and education, the ESA region’s young people still experience challenges in relation to their SRHR. This study brings together learning from the available data and reports, as well as the experiences of those closely connected with the process of affirmation/endorsement and implementation of the ESA Commitment during the first five years. For the first time, it brings together information on the political processes leading up to the affirmation/endorsement of the ESA Commitment as well as the data available for all the RAF’s indicators, in an effort to share lessons with the SRHR community regionally and globally.

Whilst challenges remain, the ESA Commitment has instigated notable progress, made possible in part by the emphasis on multisectoral collaboration of the health and education sectors nationally and regionally. Sustained political, technical, and financial investment in young people’s SRHR will ensure that the countries of the region are able to build upon these successes and deliver on their commitments to young people during this next decade of action towards the realisation of the SDGs. Beyond this, promoting the SRHR of adolescents and young people is crucial to the achievement of the long-term vision articulated in the African Union Vision 2063, which recognises the crucial part that young people’s health, rights and well-being play in harnessing the demographic dividend.

## Footnotes

* The frame of reference for this paper is young people – defined as people aged 10–24 – in line with the ESA Commitment, but different terms and age bands are used depending on the context and availability of data.

† “Adolescent” is a term that refers to people aged 10–19.

‡ Eswatini, Malawi, Tanzania, Uganda, Zimbabwe, Zambia.

§ In seven of these countries, consultation meetings with civil society organisations, UN agencies, government representatives and other stakeholders were held for the report’s development. For those five countries that did not hold meetings, data from existing reports and sources were reviewed and utilised in the report’s development.

** Eswatini, Ethiopia, Kenya, Lesotho, Madagascar, Mozambique, Namibia, South Africa, South Sudan, Tanzania, Uganda, and Zambia.

†† There is one extra indicator presented in Table 4 that appeared in the 2016 and 2019 reports but that was not in the original RAF: *“Number of countries that provide pre-service and/or in-service training programmes on the delivery of CSE”.*

‡‡ Botswana, Eswatini, Lesotho, Malawi, Namibia, South Africa, Zambia and Zimbabwe.

## Annex. Pre/post-ESA Commitment data for four countries

By way of illustration, the table below presents a subset of the ESA Commitment indicators for four countries – Kenya, Uganda, Zambia and Malawi. These data were taken from two consecutive population-level surveys in each country – one from before the ESA Commitment and one after implementation had begun. In relation to the ESA Commitment target on reducing pregnancy, three countries – Kenya, Zambia and Malawi – saw increases in the percentage of adolescent girls who had begun childbearing, whilst Uganda saw a decrease. For the target on reducing GBV, percentages of adolescent girls who think a husband is justified in hitting or beating his wife in certain circumstances, reduced in three countries but increased in one. Amongst adolescent boys, there were decreases in Kenya and Zambia, a slight increase in Uganda and a 3% increase in Malawi. The data relating to the elimination of child marriage illustrates a decrease in the percentages of girls under 18 and under 15 who were married or in union in all four countries. Finally, in relation to comprehensive HIV knowledge, slight increases or decreases in knowledge levels are reported, except in Kenya, where the reported increase was higher (7%) for both adolescent girls and boys. These country-level impact indicators provide further evidence of the work that remains to be done in the region.

**Table:**

ESA Commitment target	Indicator (DHS/MICS)	Kenya		Uganda		Zambia		Malawi	
		2008/2009	2014	2011	2016	2013/2014	2018	2010	2015/2016
Reduce early and unintended pregnancies among young people by 75%	% of adolescent girls who have begun childbearing	17.7	19.2 (2015 MIS)	23.8	19.6 (MIS)	28.5	29.2	25.6	31.5 (2017 MIS)
Eliminate gender-based violence	% of adolescents (aged 15–19) who think a husband is justified in hitting or beating his wife in certain circumstances [female]	56.6	44.5	61.8	57.5	48.6	46.5	16.4	20.7
	% of adolescents (aged 15–19) who think a husband is justified in hitting or beating his wife in certain circumstances [male]	54.1	36.7	52.2	53.0	40.6	31.5	20.7	23.7
Eliminate child marriage	% married/in union before age 18 (aged 20–24) [female]	26.4	22.9	39.7	34.0	31.4	29.0	49.6	42.1
	% married/in union before age 15 (aged 20–24) [female]	6.2	4.4	9.9	7.3	5.9	5.2	11.7	9.0
Increase to 95% the number of adolescents and young people aged 10–24 who demonstrate comprehensive HIV prevention knowledge levels	% of adolescents with comprehensive knowledge about AIDS [female]	51.2	57.7	35.6	34.8	38.9	40.5	39.5	38.9
	% of adolescents with comprehensive knowledge about AIDS [male]	42.0	49.0	40.7	40.2	42.3	38.6	44.7	43.1
